# Use of Lung Ultrasound in Cystic Fibrosis: Is It a Valuable Tool?

**DOI:** 10.3390/children11080917

**Published:** 2024-07-30

**Authors:** Alessandra Boni, Luca Cristiani, Fabio Majo, Nicola Ullmann, Marianna Esposito, Maria Chiara Supino, Paolo Tomà, Alberto Villani, Anna Maria Musolino, Renato Cutrera

**Affiliations:** 1 Pneumology and Cystic Fibrosis Unit, Bambino Gesù Children’s Hospital, IRCCS, 00165 Rome, Italy; alessandra.boni@opbg.net (A.B.); nicola.ullmann@opbg.net (N.U.); renato.cutrera@opbg.net (R.C.); 2 Academic Department of Pediatrics, Bambino Gesù Children’s Hospital, IRCCS, 00165 Rome, Italy; luca.cristiani@opbg.net (L.C.); fabio.majo@opbg.net (F.M.); marianna.esposito@opbg.net (M.E.); alberto.villani@opbg.net (A.V.); 3 Department of Emergency, Admission and General Pediatrics, Bambino Gesù Children’s Hospital, IRCCS, 00165 Rome, Italy; mariachiara.supino@opbg.net; 4 Department of Imaging, Bambino Gesù Children’s Hospital, IRCCS, 00165 Rome, Italy; paolo.toma@opbg.net

**Keywords:** lung ultrasound, cystic fibrosis, management, pulmonary exacerbations, follow-up, bronchiectasis

## Abstract

Cystic fibrosis (CF) is a multisystem disorder characterized by progressive respiratory deterioration, significantly impacting both quality of life and survival. Over the years, lung ultrasound (LUS) has emerged as a promising tool in pediatric respiratory due to its safety profile and ease at the bedside. In the era of highly effective CF modulator therapies and improved life expectancy, the use of non-ionizing radiation techniques could become an integral part of CF management, particularly in the pediatric population. The present review explores the potential role of LUS in CF management based on available data, analyzing all publications from January 2015 to January 2024, focusing on two key areas: LUS in CF pulmonary exacerbation and its utility in routine clinical management. Nonetheless, LUS exhibits a robust correlation with computed tomography (CT) scans and serves as an additional, user-friendly imaging modality in CF management, demonstrating high specificity and sensitivity in identification, especially in consolidations and atelectasis in the CF population. Due to its ability, LUS could be an instrument to monitor exacerbations with consolidations and to establish therapy duration and monitor atelectasis over time or their evolution after therapeutic bronchoalveolar lavage. On the basis of our analysis, sufficient data emerged showing a good correlation between LUS score and respiratory function tests. Good sensitivity and specificity of the methodology have been found in rare CF pulmonary complications such as effusion and pneumothorax. Regarding its use in follow-up management, the literature reports a moderate correlation between LUS scores and the type, extent, and CT severity score of bronchiectasis. A future validation of ultrasound scores specifically in CF patients could improve the use of LUS to identify pulmonary exacerbations and monitor disease progression. However, further research is needed to comprehensively establish the role of LUS in the CF population, particularly in elucidating its broader utility and long-term impact on patient care.

## 1. Introduction

Cystic fibrosis (CF) is a rare, life-limiting autosomal recessive genetic disorder caused by mutations in the gene encoding the chloride-conducting transmembrane channel known as CF transmembrane conductance regulator (CFTR) that regulates anion transport across multiple epithelia [1]. Although CF manifests as a multisystem disease, its morbidity and mortality are primarily due to bronchiectasis, small airway obstruction, thick mucus accumulation, and progressive respiratory failure [1,2].

In the management of CF, both longitudinal assessment of lung evolution damage and early diagnosis of pulmonary exacerbations (PExs) are critical [3].

A recent state-of-the-art review by experienced pulmonologists and radiologists (MASTRO) provided guidance on the use of the most commonly used imaging techniques in CF. The experts concluded that, while chest radiography (CR) is routinely used to assess both pediatric and adult patients, its use for monitoring lung damage is controversial, particularly in advanced lung disease [4,5]. On the other hand, computed tomography (CT) remains the gold standard due to its ability to assess subtle lung changes such as bronchial wall thickening, small airway disease, or air trapping [5].

In the era of highly effective modulator therapy, the CF team requires novel diagnostic tools to detect subtle changes in lung parenchyma and limit radiation exposure, particularly in the pediatric population. Although magnetic resonance imaging (MRI) overcomes the issue of ionizing radiation exposure, its lower spatial resolution and limitations in detecting small airway involvement reduce its applicability. Furthermore, its use in children necessitates cooperation and, potentially, sedation [5].

Over the past decade, analysis of artifacts of lung ultrasound (LUS) has been increasingly used in both pediatric and adult lung disease due to its relative safety and ease of performance. Among the LUS artefacts, B-lines are one of the most studied; they are non-specific sonographic findings defined as vertical hyperechoic reverberation artefacts originating from the pleural line, extending to the bottom of the screen without fading, and moving in synchrony with lung motion [6]. These artefacts are mainly caused by the decrease in air content and the increase in lung density, which reduces the acoustic mismatch between the lung and the surrounding tissues [6,7].

During dynamic observation with LUS, respiratory movements cause the pleural lines to slide back and forth, creating horizontal A-lines parallel to the pleural one [6,7].

LUS capacity to early detect pleural effusions, atelectasis, pneumothorax, and pneumonia with subpleural lung involvement is widely accepted in the literature and proposed for various chronic diseases also in children [7,8].

**Figure 1:**
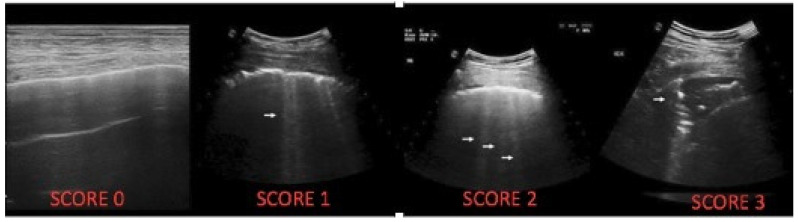
LUS CF score, score 0 = normal pattern; score 1 = scattered in clear B-line; score 2 = numerous fused B-lines; score 3 = lung consolidation. The arrow highlights the B-lines in the second and third figures, while in the fourth figure it highlights the consolidation.

In order to quantitatively assess LUS results in a standardized manner, a scoring system is used based on the evaluation of 12 lung areas. Each area is assigned a score from 0 to 3, determined by factors such as the size of consolidations, presence of bronchograms, and number of B-lines, according to the International Consensus Conference Guidelines [9] (Figure 1).

Some authors used non-validated scores (cystic fibrosis ultrasound scores) adapted to CF based on the modified Chrispin–Norman and bronchiolitis score for CF patients [10]. Other authors developed a specific CF-adapted score with distinction based on the size of consolidation and the presence of bronchogram and with relative categorization of lung damage into normal, mild, moderate, and severe [11] (Table 1).

Although validated in other contexts, the use of LUS in routine CF care remains limited [5]. To the best of our knowledge, as the literature does not report reviews on such use, our aim was to review all data available on the use of this emerging clinical tool in the management of CF to support the recognized gold-standard methods. Our objective was to analyze its possible use in CF care.

## 2. Methods

**Figure 2:**
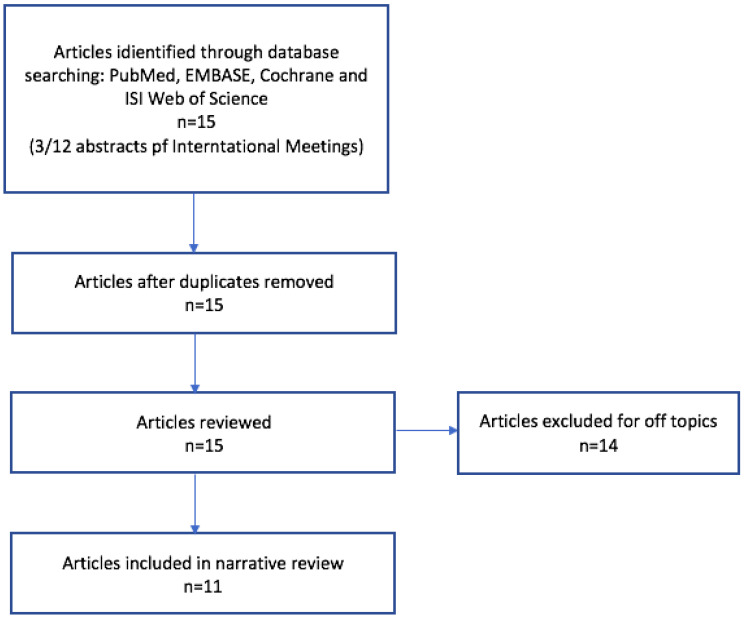
Data selection flow chart; n = numbers.

In order to conduct our narrative review, we performed a computerized literature search using PubMed, EMBASE, Cochrane, and ISI Web of Science databases: Relevant papers from January 2015 to January 2024 were selected. We reviewed studies and included available publications on both pediatric and adult populations. We considered available abstracts and posters presented at international meetings. The following search strings and medical subject headings (MeSH) terms were used individually or in combination: cystic fibrosis or lung ultrasound or pulmonary exacerbation or bronchiectasis; consolidations or atelectasis or pneumothorax or pleural effusion. Each author independently reviewed the literature for eligibility. There were no restrictions on study design or language. A cross-reference search was performed to identify any additional relevant data. Articles that did not fit into the conceptual framework of this review or did not report LUS experience with CF population were excluded (Figure 2).

We selected all 11 resulting papers reported in the literature (Table 2) and structured our manuscript by categorizing them into two main areas of interest: the role in CF pulmonary exacerbations/acute complications and in routine clinical scenarios under stable conditions. The definition of pulmonary exacerbation was established according to Fuchs’s criteria, indicated when 4 of the 11 criteria are present.

## 3. Results

### 3.1. Role in Pulmonary Exacerbations and Acute Complications

PExs are a common and potentially dangerous clinical event in the course of CF; they contribute to disease morbidity and mortality impacting lung function decline and disease progression [12]. In fact, in the case of PExs, an estimated 25–50% of patients fail to return to their baseline lung function despite an adequate treatment [3,20].

The standard definition of PExs is based on Fuchs’ criteria, which, however, are used only for research purposes and are difficult to apply in clinical practice. This definition, mainly based on clinical criteria, broadly considers any radiographic change suggestive of pulmonary infection [21]. Other groups of researchers have proposed similar criteria without radiological definitions [22].

Although limited data are available, LUS may be useful to clinicians in both the recognition and management of PExs. Preliminary data showed a good relationship between LUS and CT in detecting structural changes in the CF lung [10,17]. LUS is particularly useful in detecting lung consolidations and air bronchograms during PExs, as demonstrated by Hassanzad et al. [18]. Similarly, a 2-year prospective observational study of 82 CF patients showed a good correlation between LUS and CT in the detection of lung consolidations (r = 0.79, *p* < 0.001) [12].

The same authors also showed that LUS was better than CR both in the detection of air bronchograms and pulmonary consolidations (area under the receiver operating characteristic curve [AUROC]: 0.096 vs. 0.483 and 0.900 vs. 0.575) [18]. LUS has also been reported as capable of detecting subpleural consolidations and atelectasis as well as CT [11]. For atelectasis, a sensitivity of 83.7% and a specificity of 94.5% were reported, with a PPV of 92.5% and a NPV of 72.3%. For pulmonary consolidations, a sensitivity of 94.4% and a specificity of 93.02% was described, with a PPV of 89.4% and NPV of 97.3% [11]. They also reported similar sensitivity and specificity of LUS in identifying lung consolidation but with different predictive values (positive of 94.7% and negative of 81.8%) and with an AUROC of 0.900 (95% CI 0.766–1.000, *p* < 0.001) [18]. All data are summarized in Table 3.

A key aspect in the definition of PExs in CF is the change in forced expiratory volume in one second (FEV_1_) spirometric values, defined as a change of more than 10% change. This means that when assessing PExs in CF patients, significant changes in spirometry measurements such as FEV_1_ or forced vital capacity (FVC), exceeding a 10% decrease from baseline, are considered indicative of a pulmonary exacerbation [21].

It is noteworthy that some authors have attempted to correlate LUS score with pulmonary function.

Ciuca et al. investigated the relationship between LUS score and pulmonary function tests (PFTs) and showed a significant and strong correlation with both the lung clearance index (LCI) (rho = 0.8 with *p* = 0.0001) and a moderate correlation with FEV1 (rho = 0.65 with *p* = 0.000) and forced expiratory flow at 25–75% (FEF 25–75) (rho = 0.542 with *p* = 0.000) [11,15]. Similarly, Peixoto et al. showed a moderate correlation between LUS score and pre-bronchodilator FEV1 (rho = 0.536) [17]. The same results were reported by Curatola et al., who showed a partial correlation between LUS score and PFTs based on age and BMI [9].

Given the reported studies, we can hypothesize that LUS, together with standard PFTs, may help physicians detect PEX. In particular, it should be underlined that the strength of the correlation between LUS and LCI is stronger than that with spirometry (strong versus moderate). This could be explained by the particular capacity of LUS to detect changes in lung ventilation by identifying B-lines spectra. In fact, LCI is an early marker of ventilation inhomogeneity, reflecting early airway dysfunction in the CF population even in the presence of normal PFTs [11].

Ciuca et al. also showed a stronger correlation between LUS and LCI in CF patients with a LUS score higher than 10, consistent with severe lung morphological changes. On the other hand, the correlation was weak in patients with a mild LUS score (3–6), suggesting that LUS could be used as an early surveillance method for advanced CF lung disease and PExs [15].

As suggested by Peixoto [16] et al., a promising role for LUS could be the monitoring of patients during and after a PExs requiring intravenous antibiotic therapy. Indeed, there is still no general consensus about the optimal length of an intravenous antibiotic cycle in CF. In this scenario, LUS could help the clinical evaluation [23]. In clinical practice, the assessment of intravenous antibiotic therapy response is limited to general examination, PFTs, and blood analysis, with great limitations and loss of specificity in the case of advanced lung disease. In this case, changes in LUS score may represent another outcome of efficacy, useful for individualized therapy modulation [16].

Pleural effusion is an uncommon complication of CF. There is a limited evidence in the literature on its nature, with a reported incidence of 43 cases per 10,000 person-years in hospitalized CF patients [24]. As in other lung diseases, LUS is 100% specific for the diagnosis of pleural effusion in CF, and it is comparable to CR, as shown by Hassanzad et al. [18]. Ciuca et al. also reported the ability of LUS to detect pleural effusion as well as CF; however, the group size was very small [12].

Compared to CR, LUS is more sensitive both in detecting both low volumes of pleural fluid (<200 mL) and fluid nature [25].

Spontaneous pneumothorax occurs in up to 4% of CF patients during their lifetime, with older age and advanced lung disease being major risk factors [26]. In the literature, LUS sensitivity and specificity for pneumothorax have been reported to be 100%, although no CF patients were included [27]. A single-center, retrospective study by Scialanga et al. investigating chest pain in the emergency department showed that a radiological LUS sign called “lung point” had a sensitivity of 92.3% (95% CI 77.8–100) and a specificity of 100% (95% CI 94.4–100) for pneumothorax detection. Similarly, another specific pneumothorax LUS marker, the barcode sign, showed a sensitivity of 100% (95% CI 75.3–100) and a specificity of 100% (95% CI 94.4–100) [28].

### 3.2. Role in Follow-Up Evaluations of Stable Patients

In clinically stable CF patients, the main chest radiological findings are bronchiectasis, mucus plugging, and air trapping along with persistent atelectasis/consolidations [5].

Bronchiectasis is characterized by abnormal, irreversible bronchial dilatation, often in combination with a clinical syndrome of recurrent or persistent wet/productive cough, airway infection, and/or inflammation [29]. Radiologically, bronchiectasis is defined by three parameters: a still-recognizable bronchus up to 1 cm from the parietal pleura, the absence of narrowing, and a particular relationship to vessel caliber.

There are several degrees of bronchiectasis ranging from a mild dilatation of the bronchial wall (fusiform/cylindrical) to an almost spherical expansion of the bronchial tree (cystic bronchiectasis) [29].

CT is the gold standard for the diagnosis of bronchiectasis, especially in the early stages, and is routinely performed in CF care from an early age. The role of LUS in the assessment of bronchiectasis has not been established yet [5].

**Figure 3:**
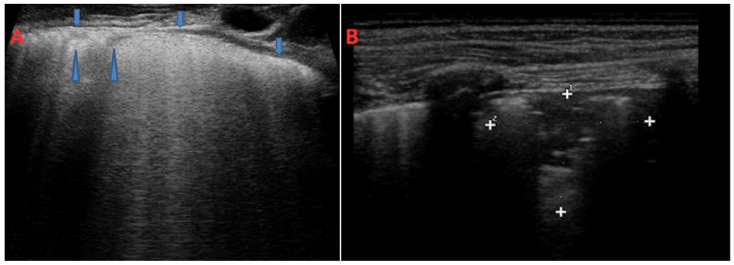
B-lines and consolidation pattern on LUS in CF patients. In Figure (**A**), a case of a 22-year-old-CF patient with B-lines pattern of coalescent B-lines and subpleural microconsolidations during a PEx. The arrows in Figure (**A**) show the subpleural hyperechogenic line and microconsolidations. In (**B**), the case of a 6-year-old CF patient with saccular bronchiectasis and consolidation patterns with air bronchogram. The ultrasound pointer circumscribes the consolidation for measurement.

A study conducted by an Egyptian group on 61 patients with non-CF bronchiectasis aimed at evaluating the diagnostic accuracy of LUS in identifying bronchiectasis compared with CT [14]. Two patterns of ultrasound abnormalities were detected: a B-line pattern in 68.8% of patients and a C-profile (consolidation) pattern in 11.1% (Figure 3). In a third small group of patients (19.7%), LUS was unable to detect any abnormalities. Regarding the type of bronchiectasis, cylindrical bronchiectasis significantly correlated with the B-lines pattern (57.1%, *p* = 0.044), whereas cystic bronchiectasis significantly correlated with C-profile (100.0%, *p* = 0.000) [14]. The same authors also demonstrated a correlation between the Reiff bronchiectasis severity score and the ultrasound patterns; in addition, the 12 patients with a modified Reiff score of less than 5.17 had normal sonography evaluation [14].

Interestingly, patients with bronchiectasis and C-pattern had a higher mean Reiff score than patients with B-pattern (9.79 versus 16.71) [14]. A significant correlation between abnormality patterns and FEV1 was also demonstrated with a higher mean FEV1 with normal sonographic examination [24].

Barakat et al. evaluated isolated LUS B-lines patterns by comparing CT and lung function data among patients with non-CF bronchiectasis [13]. All 91 patients had diffuse bilateral B-lines, and a strong correlation with the type (rho = 0.729; *p* < 0.0001) and moderate correlation with extent of bronchiectasis (rho = 0.640; *p* < 0.0001) on chest CT was found. In addition, the number of B-lines was inversely but weakly correlated with Tiffenau index (rho = −0.281, *p* < 0.007), FEV1 (rho = −0.339, *p* < 0.001), and FEF 25% to 75% (rho = −0.389, *p* < 0.000) but moderately correlated with arterial partial pressure of oxygen (rho = −0.612, *p* < 0.000) [13].

Conversely, others authors pointed out that LUS was able to intercept all saccular bronchiectasis in all 82 patients, whereas tubular bronchiectasis escaped LUS more easily and showed a low LUS/CT correlation (rho = 0.14, *p* < 0.9) [12].

The same authors investigated the sensitivity and sensibility of LUS in bronchiectasis in a group of 57 CF patients and demonstrated a variability in the accuracy according to the type of bronchiectasis. They showed a relatively good sensitivity in the detection of cylindrical bronchiectasis (77.7%) and saccular bronchiectasis (68.4%), but a low specificity for cystic bronchiectasis (9%) was observed [11]. They concluded that multiple B-lines may represent not only interstitial inflammatory lesions but also small bronchiectasis in CF patients [11].

Another main goal in the follow-up of stable CF patients is the analysis of the progression of chronic structural damage. Useful tools for this purpose are radiological scores, which play a crucial role in the evaluation.

Ciuca et al. also demonstrated a strong correlation between the modified Bhalla severity CT score and the LUS-CF score (rho = 0.87, *p* = 0.000) in the CF population, suggesting a good reliability in the evaluation of lung parenchymal deterioration, especially in moderate lung disease (rho = 0.57, *p* = 0.01) [11]. In a 2-year prospective observational study of 82 CF patients, the same author demonstrated an age-dependent increase in the B-line (42.6%), with a greater involvement in older patients [13]. Jaworska et al. described a moderate correlation between the number of Am-lines and the modified Chrispin–Norman CF-specific radiological score for lesions consistent with bronchiectasis on CRs in CF patients [29]. Am-lines are broad vertical artefacts consisting of multiple parallel horizontal artefacts ending at the bottom of the screen, resulting from multiple reflections of the ultrasound waves between two surfaces, the pleural line, and the wall of emphysematous bulla/cyst or bronchiectasis [19].

LUS-CF score seemed to represent well the categories of mild, moderate, and severe lung damage according to Bhalla’s score and functional impairment [11].

Furthermore, the LUS score seems to correlate not only with lung function but also with the microbiological status, which is higher in chronically colonized patients and especially in those with fungal infection, as demonstrated by other authors [29].

Pleural irregularity or thickening can be detectable by LUS in CF patients. However, no study has shown a significant correlation with other radiographic techniques, mainly because of the lack of agreement with specific findings on CT and CR [10,15].

Regarding air trapping and mucus plugging, LUS does not seem to able to evaluate these changes due to the lack of LUS-specific artefacts [11,29]. Regarding bronchial thickening, low sensitivity and specificity of 31.7% and 35.2%, respectively, were reported [11].

In Table 4, we summarize the results regarding the role of LUS in the follow-up of stable CF patients.

## 4. Discussion

We conducted this narrative review of the literature to assess the validity of an emerging tool in clinical practice. The simplicity of the procedure and the absence of ionizing radiation make it a practical and versatile tool in clinicians’ hands.

The good correlation between LUS and CT and the high sensitivity for the detection of consolidations and atelectasis make it a useful tool for the diagnosis and management of PExs with these radiological findings [12,15,17]. The use of LUS, in combination with clinical and spirometric changes according to Fuch’s criteria, could be useful in detecting a PEx [9,29]. Another strength of LUS in PExs could help the clinician detect PExs and, therefore, determine the duration of antibiotic therapy [19]. In fact, FEV1 monitoring is a valid control point for defining PExs and determining the need to change or prolong the antibiotic therapy up to 21 days.

However, currently, LUS does not outperform other radiological methods in the follow-up of stable CF patients, but it can be very useful in detecting the evolution of parenchymal damage. In fact, the LUS-CF score shows a very good correlation with modified Bhalla CT score and Chrispin–Norman CR score [13,14]. The availability of a radiological score that is simple to obtain and is repeatable over time can be useful to the clinician for understanding the progression of the patient’s lung disease or establishing the execution timing of other radiological investigations (e.g., CT or MRI) in declining CF patients. Furthermore, the LUS score is simple and quick to determine by the operator during the examination, while other radiological scores are more burdensome and, therefore, now rarely used in clinical practice.

A current limitation in the use of ultrasound scores is the lack of validation in the CF population. Papers in the literature report different scores modulated on the basis of Chrispin–Norman or even bronchiolitis, making comparison difficult [10]. However, in the same way that Fuch’s criteria highlight changes in clinical and radiological features, we believe that the score should be used for detecting changes in these features. For example, a change from mild to moderate score could be a sign of PExs or disease progression, which is associated with clinical assessment and PFTs in the timeline evaluation of a chronic disease.

Another limitation of the methodology is its lack of specificity in patients with a milder spectrum of the disease (e.g., young children, children, and adolescents under highly effective modulator therapy), where the only sign of radiological disease might be air trapping that LUS can easily identify.

However, as LUS is a relatively new method, as is the advent of highly effective CFTR modulator therapy, we believe further studies are needed. In particular, it would be useful to compare LUS images of patients with mild lung disease with CT images. Presumably, mucus plugging and air trapping could be represented by a particular presentation of B-lines or pleural line irregularity. In fact, as reported for other diseases, pleural line irregularity could reflect the exaggerated accumulation of air in the lungs due to the mucus plugs [30].

Additionally, in the era of highly effective CFTR modulators, we should start discussing radiological dose reduction, and LUS could be a valid tool to extend the timeline.

On the other hand, in some cases, LUS cannot accurately characterize the nature of damage; for example, it may struggle to distinguish between inflammation and lung fibrosis, requiring the operator’s skills and experience to detect specific pulmonary changes. It is also important to note that detectable artefacts do not exhibit enough specificity. In fact, LUS artefacts may represent different pathological expression; for example, B coalescent lines can be suggestive of alveolo-interstitial inflammation, mucus plugging with loss of aeration, or bronchial wall thickening.

Despite these limitations, it might be useful to include a routine LUS in the follow-up of stable patients in order to limit radiation exposure, longitudinally assessing LUS score changes given the close correlation with other radiological methods. We can assume that LUS can be performed in all cases of suspected PExs and at least once a year to monitor parenchymal damage, as it could be a valuable tool in care of the CF population, especially in the novel modulator era.

## 5. Conclusions

According to the data of our research, we believe that LUS may have a role in clinical practice in CF, especially for identifying pleural effusion, pneumothorax, consolidations in PExs and monitoring atelectasis. In the latter scenario, LUS could be a tool to control the effect of broncholavage with mucolytic solutions or the effect of respiratory physiotherapy. On the other hand, in the case of PExs with consolidations, LUS could be a tool to decide the duration of intravenous antibiotic therapy, which is still debated in CF.

We suggest that the LUS score should be validated in the CF population to detect sonographic changes. By analyzing the change in scores associated with clinical features and PFTs, we could intercept PExs or progression of parenchymal damage of the disease, given the correlation with CT. In this perspective, LUS would become a valid tool to reduce the dose of radiological exposure.

In particular, a future research strategy could be to analyze the delta of change in LUS score in CF exacerbations defined by Fuchs’ criteria. By doing so, as with spirometry decline, a cut-off could be identified.

Ultimately, more data are needed to assess the LUS role in all stages of CF lung disease, such as in PExs/acute complications and in routine follow-up settings. Based on the current literature, we might consider LUS as an additional valuable, easy-to-perform tool along with standards of care in the assessment of CF patients.

## Figures and Tables

**Table 1:** LUS-CF artefacts score.

LUS Artefact	Lung CF Score
Presence of A lines-normal aspect; distinctive B-lines < 3/ic space	0
Distinctive B-lines > 3/space or 1 coalescent B-line	1
Coalescent B-lines > 2/ic space	2
Consolidation < 1 cm	3
Consolidation > 1 cm, with bronchogram	4
Atelectasis/consolidation without bronchogram, >1 cm	5

**Table 2:** All selected papers for revision. CT = computed tomography; CR = chest radiograph; PExs = pulmonary exacerbations.

	Country	Population Size	Age Categories	Setting	Main Results
Curatola et al., 2023 [9]	Italy	29	Pediatric and adults	Outpatient	Correlation between LUS score and spirometric values
Strzelczuk–Judka L et al., 2019 [10]	Poland	48	Pediatric	Outpatient	Correlation of LUS with CT, detection of subpleural consolidation higher than CR
Ciuca et al., 2022 [11]	Romania	98	Pediatric	Outpatient	Correlation of LUS with CT, higher sensitivity and specificity to detect atelectasis and consolidations
Ciuca et al., 2016 [12]	Romania	82	Pediatric and adults	Outpatient	Correlation of LUS with CT
Barakat M et al., 2016 [13]	Egypt	91	Not specified	Outpatient	Correlation between number of B-lines with type and extent of bronchiectasis
Ghany MFA, 2019 [14]	Egypt	61	Adult	Outpatient	Correlation of LUS with severity of bronchiectasis CT score
Ciuca et al., 2018 [15]	Romania	42	Not specified	Outpatient	Correlation of LUS with LCI
Peixoto AO et al., 2019 [16]	Brazil	2	Adults	Hospitalization	Use of LUS score to monitor end of antibiotic intravenous therapy
Peixoto AO et al., 2020 [17]	Brazil	18	Adults	Outpatient	Correlation of LUS with CT, functional test and nutritional status
Hassanzad M et al., 2021 [18]	Iran	30	Pediatric and adults	Hospitalization	LUS is superior to CR and comparable with CT in PExs
Jaworska J et al., 2023 [19]	Poland	131	Pediatric	Outpatient	Correlation of LUS with CR, pulmonary function, and microbiological status

**Table 3:** Role of LUS in PExs and acute complications: state of evidence in the literature. n = number of patients; R = rho by Spearman; *p* = *p*-value; PPV = positive predictive value; NPV = negative predictive value; AUROC = area under the receiver operating characteristic curve.

	Patients n	LUS vs. CXR	LUS vs. CT	Consolidations	Pleural Effusion	Interstitial Syndrome	Atelectasis
Peixoto AO et al. [17]	18	/	R = 0.607 *p* = 0.001	/	/	/	/
Strzelczuk–Judka L et al. [10]	48	R = 0.52 *p* = 0.0002	/	/	/	/	/
Hassanzad M et al. [18]	30	[AUROC] = 0.900 vs. 0.575	/	Specificity 90% Sensitivity 94.7% PPV 94.7% NPV 81.8%	Specificity 96.7% Sensitivity not calculated PPV 0% NPV 100%	/	Specificity 100% Sensitivity not calculated PPV 93.3% NPV not calculated
Ciuca, I et al. [12]	82	/	R = 0.79 *p* < 0.0001	/	/	/	/
Ciuca I et al. [15]	57	/	R = 0.87 *p* = 0.000	Specificity 93.02% Sensitivity 94.4% PPV 89.4% NPV 97.3%	/	/	Specificity 94.5% Sensitivity 83.7% PPV 92.5% NPV 72.3%

**Table 4:** LUS role in the follow-up bronchiectasis in CF stable patients. n = number of patients; R = rho by Spearman; *p* = *p*-value; PPV = positive predictive value; NPV = negative predictive value.

	Patients n	LUS vs. CT	Saccular/Cystic	Tubular	Cylindrical
Ciuca, I et al. [12]	82	/	PPV 100%	R = 0.14 *p* < 0.9	/
Ciuca I et al. [15]	57	/	Sensitivity 68.4% Specificity 94.9% PPV 88.8% NPV 94.7%	/	Sensitivity 77.7% Specificity 9% PPV 80.7% NPV 76.9%
Ghany MFA et al. [14]	91	Correlated with severity of bronchiectasis (by modified Reiff score pattern, *p* < 0.000)	/	Correlated with consolidation pattern (100.0%, *p* = 0.044)	Correlated with B-lines pattern (57.1%, *p* = 0.001)
M. Barakat et al. [13]	91	Correlated with type (R = 0.729; *p* < 0.0001) and extent (R = 0.640; *p* < 0.0001)	/	/	/

## Data Availability

Data available in a publicly accessible repository.

## Abbreviations

CF	Cystic fibrosis
CR	Chest radiograph
CT	Computed tomography
MRI	Magnetic resonance imaging
LUS	Lung ultrasound
PExs	Pulmonary exacerbations
PFTs	Pulmonary function tests
LCI	Lung clearance index
FEV_1_	Forced expiratory volume in 1 s
FEF 25–75	Forced expiratory flow at 25–75%
